# Overexpression of ANXA3 is an independent prognostic indicator in gastric cancer and its depletion suppresses cell proliferation and tumor growth

**DOI:** 10.18632/oncotarget.13493

**Published:** 2016-11-22

**Authors:** Ke Wang, Jiansheng Li

**Affiliations:** ^1^ Department of Gastroenterology, The First Affiliated Hospital of Zhengzhou University, Zhengzhou 450052, Henan, China

**Keywords:** gastric cancer, ANXA3, proliferation, cell growth, survival

## Abstract

**Background:**

Gastric cancer (GC) is one of the most common malignancies worldwide. Tumour metastasis is one of the leading causes of death in GC patients. This study aims to investigate the significance of ANXA3 expression and the mechanism by which ANXA3 is involved in the epithelial–mensenchymal transition (EMT) of gastric cancer cells.

**Results:**

Our results confirmed that ANXA3 was high expression at the mRNA and protein level in GC cancer tissues and the majority of GC cell lines. In clinicopathological analysis, we found that increased expression of ANXA3 in tumors was closely associated with a poor prognosis. Xogenous ANXA3 transduction promoted proliferation, clone formation, migration, and invasion. Small interfering RNA silencing of ANXA3 inhibited these processes. Silence of ANXA3 inhibited tumorigenicity in vivo. Additionally, ANXA3 expression is associated with the epithelial–mesenchymal transition.

**Methods:**

Firstly, we investigated the ANXA3 expression on mRNA and protein level with RT-PCR and Western blot. Secondly, 183 GC patients tissues were used the to evaluate the clinicopathological characteristics and prognosis through immunohistochemistry. Furthermore, The functions of ANXA3 were analyzed in the cell proliferation, Colony Formation, migration, invasion and apoptosis of GC cell lines.

**Conclusions:**

Our research suggests that ANXA3 plays important roles in gastric cancer carcinogenesis and metastasis, and provides a valuable prognostic marker and potential target for treatment of gastric cancer patients.

## INTRODUCTION

Gastric cancer (GC) is the third common malignancies in China, accounting for about four hundred thousand new cases each year [1]. Over the past decades, in spite of observable advancement in surgical technique, chemotherapy and radiotherapy, the overall survival for GC has not been significantly improved [2]. Tumour metastasis is a major lethal cause in GC patients [2]. It has been known that the tumorigenesis and progression of GC is fully convinced as a multi-step procedure that related to the mobilization of oncogenes and the restrain of tumor suppressors [3–4]. Therefore, to understand the molecular mechanisms governing the carcinogenesis and metastasis of GC is anticipated.

The epithelial–mesenchymal transition (EMT), an essential process in development of embryos, is involved in the metastasis and progression of tumors [5–7]. Activation of the EMT program, along with an accumulation in expreission of the mesenchymal marker vimentin and loss of the epithelial marker E-cadherin, endows carcinoma cells with enhanced migratory and invasive capacities that facilitate dissemination to permissive niches [8]. EMT is a process initiated by a series of cumulative genetic and epigenetic modifications that occur in transforming cells and signals from the tumor microenvironment [9]. All sorts of signaling pathways are participated in EMT, including the tumor growth factor-β(TGF-β), nuclear factor-kB (NF-kB), RTK/Ras and Wnt/β- catenin pathways, correlated with tumor progression [9–12].

Annexin A3 (Anxa3), as well as designated as lipocortin3, placental anticoagulant protein3 (PAP-III), subordinate to annexin family [13]. It has three different domain composition: a short The C-terminus domain including four conserved ‘annexin repeat’ domains I, II, III and IV, a repeated collagen-like sequence, and a COOH-terminal domain including a single carbohydrate recognition-binding domain [14–15]. It is a membrane associated protein involved in the regulation of varieties of biological responses, containing cell proliferation, angiogenesis, cancer progression and metastasis [16–18]. ANXA3has been identified as a putative tumor oncogene in a diversity of carcinomas, such as Ovarian carcinoma, Lung adenocarcinoma, hepatocellular carcinoma, Colorectal cancer [19–22]. Overexpression of ANXA3 significantly promoted tumor cell growth and increased the tumorigenic potential of a number of tumor lines in nude mice [21]. However, the role of ANXA3 in GC has not been further investigated.

The findings in this report provide proof that ANXA3 is unregulated in GC cell lines and tumor tissue samples. Further evidences illustrate that enforced ANXA3 expression is linked with poor prognosis in GC. We show that ANXA3 promotes GC proliferation, colony Formation, migration and invasion in vitro. We further demonstrate that ANXA3 expression is associated with the epithelial–mesenchymal transition in this study. Our findings indicate that the ANXA3 may serve as a latent target for diagnosis and therapy in GC.

## RESULTS

### ANXA3 was up-regulated in GC tissues and cell lines

**Figure 1:**
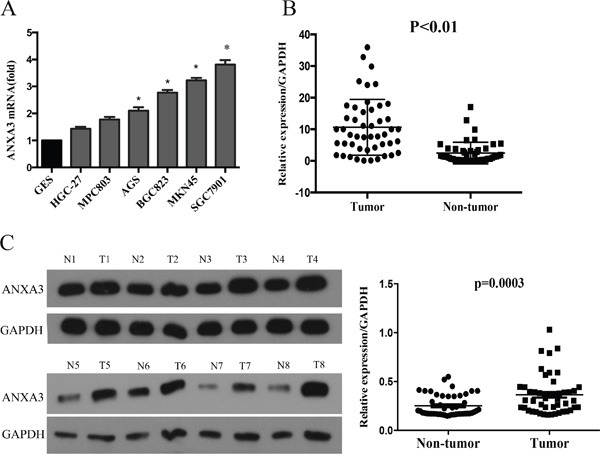
**A.** The mRNA level of ANXA3 was examined in gastric cancer cell lines using RT-PCR. **B**. The mRNA level of ANXA3 was examined in gastric cancer tissues and Normal tissue using RT-PCR. *P<0.05 vs. control. **C**. The protein level of ANXA3 was determined using western blot analysis in gastric cancer tissues and Normal tissues.

In this research, ANXA3 expression was analyzed in GC tissues and six GC cell lines MKN45, SGC7901, MGC803, BGC823, AGS, and HGC27. RT-qPCR assay illustrated that in comparison with the normal cell line GES, ANXA3 was markedly higher in the GC cell lines (Figure 1A). RT-qPCR assay and Western blotting indicated that ANXA3 were upregulated in GC tissues compared to the normal adjacent tissues (Figure 1B and 1C). These results suggest that upregulation of ANXA3 is involved in Gastric cancer.

### ANXA3 expression is an independent prognostic factor for patient survival

**Figure 2:**
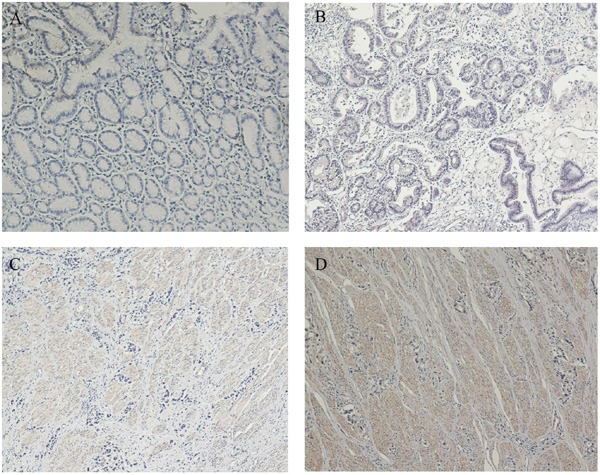
ANXA3 protein expression in gastric cancer surgical specimens shown by immunohistochemistry **A**. ANXA3-negative staining was observed in noncancerous gastric mucosa. **B**. High ANXA3 staining was observed in gastric cancer tissues. **C**. Moderate ANXA3 staining was observed in gastric cancer tissues. **D**. Poorly ANXA3 staining was observed in gastric cancer tissues.

**Figure 3:**
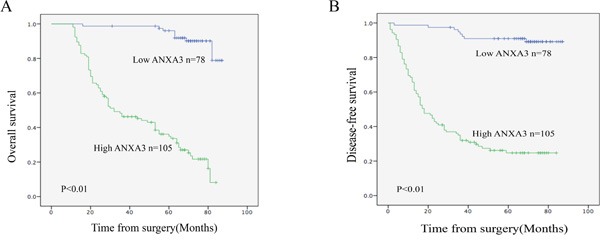
Kaplan-Meier survival curves of gastric cancer patients after gastrectomy **A**. The overall survival of patients in the ANXA3-high group was significantly lower than that of patients in the ANXA3-low group (log-rank test, n=183, P<0.01). **B**. The Disease-free survival of patients in the ANXA3-high group was significantly lower than that of patients in the ANXA3-low group (log-rank test, n=183, P<0.01).

To further investigate the correlation between ANXA3 expression and clinicopathological features, along with the prognostic role of ANXA3 in GC, 183 paraffin-embedded gastric cancer tissue samples were used for immunohistochemical analysis. Firstly, we determined expression of ANXA3 in 183 gastric cancer tissue samples (Figure 2), and divided these patients into two staining: high ANXA3 staining (n=105) and low ANXA3 staining (n=78). Analysis of clinicopathological parameters showed that high ANXA3 expression was striking related to Tumor size (P=0.006), depth of tumor infiltration (T stage, P<0.001), TNM stage (P<0.001), and the local lymph node metastasis (N stage, P=0.023), Distant metastasis(P=0.014) (Table 1). Kaplan-Meier survival analysis indicated that patients with low ANXA3 expression had longer OS and DFS (P<0.001, Figure 3A and 3B). Furthermore, univariate analysis implied that overall survival was significantly correlated with ANXA3 expression (P < 0.001), depth of tumor infiltration(P = 0.027), TNM stage (P<0.001) and the local lymph node metastasis (N stage, P=0.012), Distant metastasis((P < 0.001), but not with sex, age, Tumor size (Table 2). Multivariate analysis of these parameters indicated that ANXA3 served as a beneficial prognostic factor for GC patients.

**Table 1:** Correlation between ANXA3 expression and clinicopathological variables of 183 gastric cancer cases

Clinicopathological parameters	*n*^a^	ANXA3 Expression		*χ*^2^	*P* Value
		High	Low		
**All**	183	105	78		
**Age (years)**					
<55	85	43	42	2.991	0.084
≥55	98	62	36		
**Gender**				0.073	0.787
Male	66	37	29		
Female	117	68	49		
**Tumor size**				7.627	0.006*
<3 cm	36	28	8		
≥3 cm	147	77	70		
**Tumor infiltration**				23.649	<0.001*
T1	29	25	4		
T2	20	17	3		
T3	14	6	8		
T4a	93	47	46		
T4b	27	10	17		
**Local lymph node metastasis**				9.534	0.023*
N0	59	38	21		
N1	34	21	13		
N2	35	12	23		
N3	55	34	21		
**Distant metastasis**				6.242	0.014*
M0	164	89	75		
M1	19	16	3		
**TNM staging**				20.398	<0.001*
I	37	33	4		
II	34	14	20		
III	93	48	45		
IV	19	10	9		

^a^Numbers of cases in each group. * Statistically significant (*P*<0.05).

**Table 2:** Univariate and multivariate analyses of overall survival of gastric cancer patients

Variables	*n*^a^	Univariate analyses			Multivariate analyses		
		HR	(95% CI)	*P* Value	HR	(95% CI)	*P* Value
**Age (years)**				0.176			
<55	85	1.000					
≥55	98	1.589	0.979-2.019				
**Gender**				0.270			
Female	66	1.000					
Male	117	1.503	0.946-2.217				
**Tumor size**	0.001*	0.367					
<3 cm	36	1.000	1.000				
≥3 cm	147	6.327	3.015-9.367	2.352	0.980-4.137		
**Tumor infiltration**	<0.001*	0.027*					
T1	29	1.000	1.000				
T2	20	3.247	1.058-5.719	2.319	1.007-5.197		
T3	14	5.197	3.033-7.184	3.019	1.195-6.886		
T4a	93	8.119	5.157-12.679	4.691	2.119-8.793		
T4b	27	8.915	6.217-14.398	5.138	3.325-9.873		
**Local lymph node metastasis**	<0.001*	0.012*					
N0	59	1.000	1.000				
N1	34	2.105	1.035-5.273	1.245	1.137-2.566		
N2	35	4.376	2.286-6.713	3.579	1.318-5.895		
N3	55	6.336	3.281-7.635	5.521	2.377-9.863		
**Distant metastasis**	<0.001*	<0.001*					
M0	164	1.000	1.000				
M1	19	5.875	3.345-9.123	2.431	1.527-6.318		
**TNM staging**				<0.001*			<0.001*
I	37	1.000			1.000		
II	34	2.165	1.004-4.381		2.345	1.022-5.541	
III	93	5.217	2.094-7.299		2.647	1.227-5.775	
IV	19	6.332	3.191-9.507		4.488	2.191-8.507	
**ANXA3**				<0.001*			0.005*
Low	78	1.000			1.000		
High	105	5.425			3.279-10.647	4.518	2.328-8.818

HR, hazard ratio; CI, confidence interval; aNumbers of cases in each group; * Statistically ignificant (*P* < 0.05).

### ANXA3 promotes cell growth of GC cells

**Figure 4:**
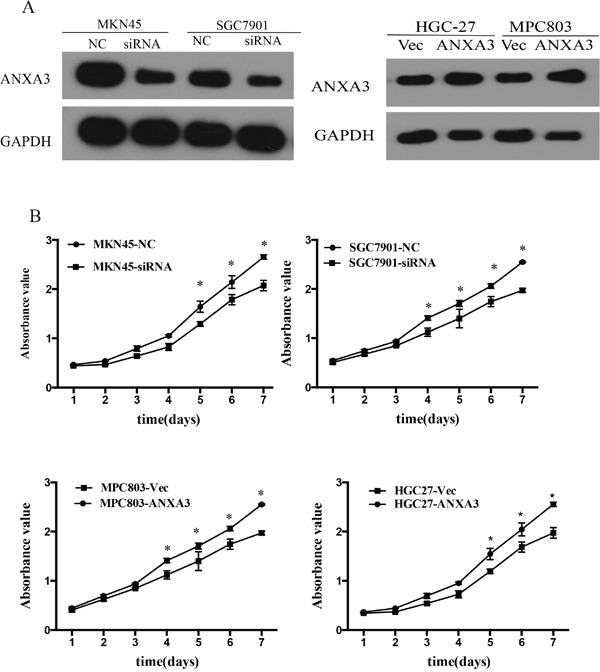
**A**. The expression of ANXA3 protein in SGC7901 and MKN45 cells infected with siRNA, HGC-27 and MGC803 cells infected with Ad-ANXA3 and Control. **B**. MTS assays were performed to compare the cell growth rates between ANXA3-overexpressing and control cells; between ANXA3-silenced and control cells.

**Figure 5:**
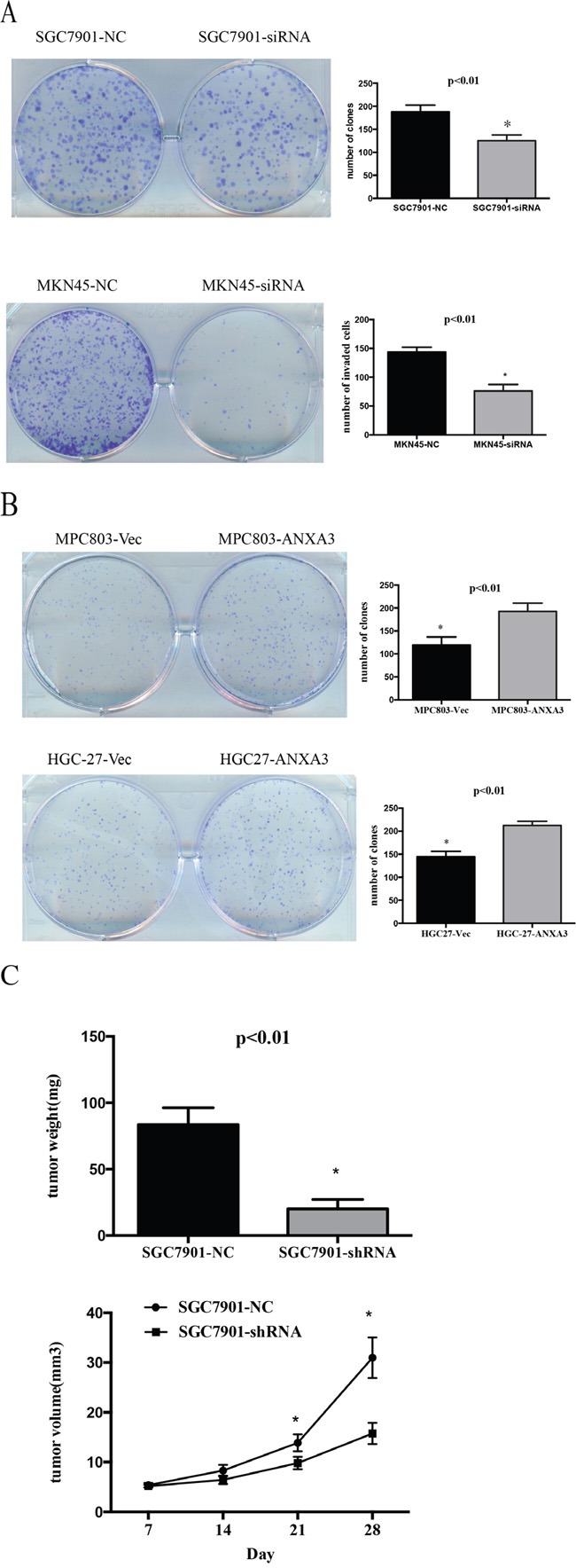
**A-B**. Representative images of the increased colony formation ability induced by ANXA3 in gastric cancer cell lines. Quantitative analyses of colony formation numbers are shown in the right panel. **C**. Representative images of xenografts and a summary of tumor weight in nude mice. The weights of xenograft tumors are summarized in the right panel. All results are expressed as the mean ± SD of three independent experiments. *, P<0.05.

The SGC7901 and MKN45 cells with high ANXA3 expression were infected with ANXA3 siRNA and negative control. The MPC803 and HGC27 cells with low ANXA3 expression were infected with ANXA3 overexpression Lentivirus and control. Western blotting confirmed ANXA3 expression in the infected cells (Figure 4A). The MTS assays illustrated that ANXA3 overexpression accelerated the growth of MPC803 and HGC27 cells, whereas ANXA3 knockdown inhibited the growth of the SGC7901 and MKN45 cells (P<0.05, Figure 4B). Colony formation assays revealed that ANXA3 siRNA cells reduced the number and size of colonies compared to the control cells. ANXA3-overexpressing cells yielded more and larger colonies compared with control cells (P<0.05; Figure 5A-5B). To investigate the role of knockdown ANXA3 in vivo, we generated SGC7901subline with stably knockdown ANXA3 and a control cell line were injected subcutaneously into the flanks of nude mice. The volumes and weights of the tumors of the ANXA3 group were significantly smaller than those of the control group (Figure 5C).

### ANXA3 promotes migration and invasion of GC cell

**Figure 6:**
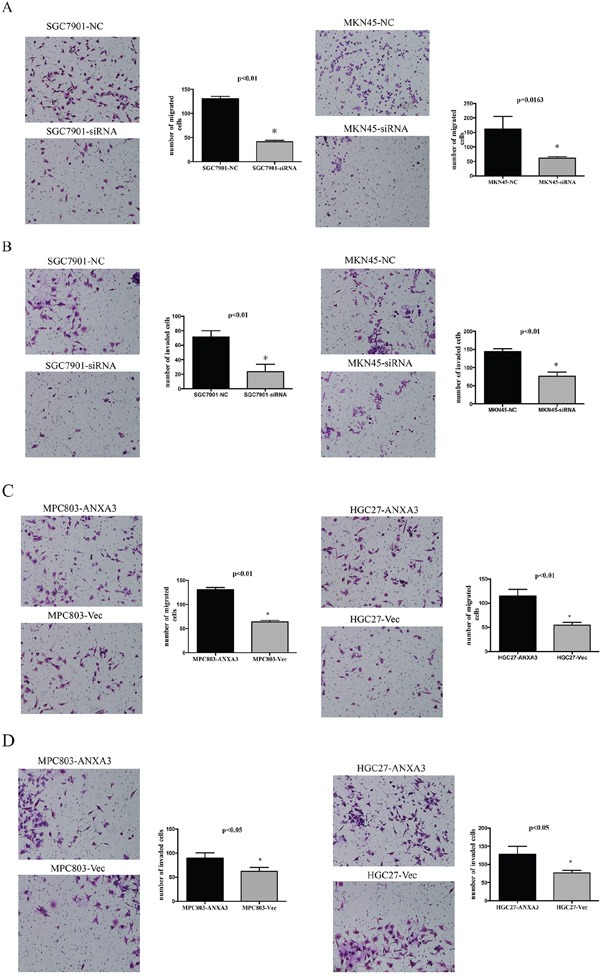
ANXA3 promoted migration and invasion of gastric cancer cells ANXA3 significantly promoted the migration **A**. and invasion **B**. of MGC803 and HGC-27 cells expressing high level of ANXA3 compared with control vector. ANXA3 significantly inhibited the migration **C**. and invasion **D**. of MKN45 cells and SGC7901 cells transfected with siRNA compared with NC. The values shown are expressed as the mean ± SD of three independent experiments. *, P<0.05 versus control.

To investigate ANXA3 function of cell migration and invasion, transwell assays were assessed. Compared with the control cells, the silence of ANXA3 expression induced decrease in the migration of GC cell lines (Figure 6A). According with the migration assay, invasion assay showed that the knockdown of ANXA3 inhibited cell invasion. The invasiveness of ANXA3-overexpressing cells was significantly higher compared with controls (Figure 6B). These results indicated that ANXA3 facilitated migration and invasion of GC cells.

### ANXA3 is associated with the epithelial–mesenchymal transition

**Figure 7:**
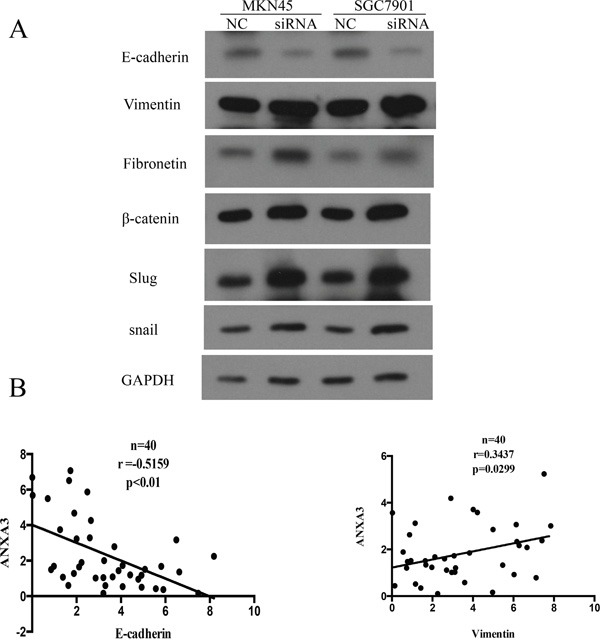
**A.** Western blot analysis was performed to determine the protein expression of EMT pathway in HGC-27 and MGC803 transfected with ANXA3 overexpression Lentivirus and MKN45 and SGC7901 cells transfected with ANXA3 small interfering RNA. **B**. Analysis of RT-PCR data showing linear regressions and significant Pearson correlations of ANXA3 with vimentin (n=40), ANXA3 with E-cadherin (n=40) in gastric cancer tissues.

To explore the relationship of ANXA3 and EMT, we investigated the protein levels of several EMT markers in GC cell lines. Western blot confirmed that ANXA3 could decrease epithelial markers (E-cadherin) and increase mesenchymal markers (vimentin and β-catenin), as well as EMT-related transcription factors (snail, fibronectin and slug) (Figure 7A). To confirm this finding, real-time PCR of ANXA3, vimentin, and E-cadherin was performed in 40 primary human GC tissue samples. The results indicated that the level of ANXA3 positively associated with level of vimentin (r=0.3437, P=0.0299; Figure 7B), and inversely correlated to level of E-cadherin (r=-0.5159, P<0.01; Figure 7B). All these findings suggest that ANXA3 is correlated with EMT markers or EMT-related transcription factors.

## DISCUSSION

ANXA3, a member of annexin family, which was a highly expressed membrane associated protein, important for the diagnosis and prognosis. Overexpression ANXA3 is associated with cancer [23–24]. ANXA3 has been reported that increased in HCC [24] and as a potential target for immunotherapy of liver cancer stem-like cells [25]. Collectively, previous findings demonstrate that ANXA3 has an important impact on the migration, invasion, and metastasis of malignant tumors [24–25]. However, the function of ANXA3 in GC has largely unknown in the literature.

Previous studies have demonstrated that annexin family members, notably ANXA3, played a key role in tumor [23, 25]. Nevertheless, there was less report involving the place of ANXA3 in GC. In our findings, we first confirmed that ANXA3 was dramatically higher in GC tissues and cell lines. The mRNA and protein level of ANXA3 were strikingly increased in most clinical GC samples. We investigated the correlation of ANXA3 in 183 paraffin-embedded GC tissue samples. We found that ANXA3 was markedly associated with Tumor size, depth of tumor infiltration (T stage), TNM stage, the local lymph node metastasis, Distant metastasis and prognosis of GC patients. These findings further represent that ANXA3 functioned as a tumor oncogene in GC.

To further elucidate the tumor oncogene role of ANXA3 in GC, we investigated the effect of ANXA3 on the viability of GC cells in vitro. Results showed that knockdown of ANXA3 expression in GC cells significantly suppressed cell proliferation and efficiency of colony formation. These results further confirmed the oncogene role of ANXA3 in GC and suggest the important function of ANXA3 in GC tumourigenesis. A study by Bianchi C indicated that ANXA3 is participated in Renal cell carcinoma metastasis through stimulating angiogenesis by increased VEGF level promoted by HIF-1a [26–27]. Pan et al found that knockdown of ANXA3 expression significantly inhibited cell proliferation and tumorigenesis in Primary Hepatocellular Carcinoma [23]. Yan et al reported that overexpression of ANXA3 might inhibited the level of p53, and thus enhanced the platinum-drug resistance in ovarian cancer cells [19, 27]. In addition, some studies demonstrated the tumor oncogene status of ANXA3 in Colorectal carcinoma, pancreatic carcinoma, and Prostatic carcinoma [28–29]. These researches, together with ours, suggest that ANXA3 serve as a tumor oncogene in a wide range of common human tumor types.

Moreover, we explored the effect of ANXA3 in cell migration and invasion in GC cells. Results showed that the facility of cell migration and invasion were significantly reduced when ANXA3 was silence. These results suggest that ANXA3 may promoted GC cell metastasis. Consistent with our findings, Pan et al reported that silence of ANXA3 significantly inhibited hepatocellular carcinoma cancer cell invasion and migration [23]. These researches demonstrate that ANXA3 may have important impact on the progression and metastasis of different human cancer types.

However, the specific mechanism involved in ANXA3 promoting gastric cancer cell metastasis has not been clarified. In our findings, we found that silence of ANXA3 dramatically inhibits the growth and migration of GC cells. Previous research has reported that ANXA3 promoted cell proliferation and migration by involving in several signaling pathway [24, 30]. For example, Tong et al. found that, enforced annexin A3 (ANXA3) enhanced cancer and stem cell-like properties in CD133+ liver CSCs by decreasing the activity of JNK [30]; Pan et al. reported that ANXA3 up-regulation in human Hepatocellular Carcinoma (HCC) may be a new prognostic biomarker, as ANXA3 promoted Hepatocellular Carcinoma cell proliferation, colony formation and migration [24]. In accordance with these previous results, we verified that ANXA3 regulated gastric cancer cell proliferation, migration, and invasion, identifying an attractive strategy to treat in gastric cancer. Additionally, ANXA3 expression can also mediate the level of vimentin, which not only is a marker of EMT but also has an important role inregulating cellular migration.

In conclusion, our study shows that ANXA3 plays a pivotal role in regulating GC metastasis by way of enhancing cellular migration, cellular invasiveness, and vimentin expression level. Moreover, a high level of ANXA3 expression can potentially be used to predict shorter OS and DFS of GC patients. ANXA3 acts as a potential prognostic factor and potential therapeutic target of GC patients.

## MATERIALS AND METHODS

### Cell lines and culture conditions

The gastric cancer cell lines, MKN45, SGC7901, MGC803, AGS, and HGC27 were obtained from the Committee of Type Culture Collection of Chinese Academy of Sciences (Shanghai, China). All cells were cultured in RPMI 1640 media supplemented with 10% heat-inactive fetal bovine serum (FBS). The cells were incubated at 37°C in a humidified chamber containing 5% CO_2_.

### Patient information and tissue specimens

This study was conducted on a total of 183 paraffin-embedded GC samples, which were histopathologically and clinically diagnosed at from 2001 to 2006. This study was approved by the Ethics Committee of The First Affiliated Hospital of Zhengzhou University. Written informed consent was obtained from each patient. Forty-eight gastric cancer specimens and the matched adjacent noncancerous tissues were frozen and stored in liquid nitrogen until further use.

### Immunohistochemistry

Immunohistochemical (IHC) analysis was used to study ANXA3 protein expression in 183 human paraffin-embedded GC samples. The procedure was carried out similarly to previously described methods [7]. The degree of immunostaining of formalin-fixed, paraffin-embedded sections was reviewed and scored independently by 2 observers, based on both the proportion of positively stained tumor cells and the intensity of staining.

### RNA extraction, reverse transcription (RT) and real-time PCR

Total RNA from cultured cells was extracted using the Trizol reagent (Invitrogen, Carlsbad, CA) as the manufacturers instruction. cDNAs were amplified and quantified in ABI Prism 7500 Sequence Detection System (Applied Biosystems, Foster City, CA) using GoScriptTM Reverse Transcriptase (Promega, Beijing, China). Expression data were normalized to the geometric mean of housekeeping gene GAPDH to control the variability in expression levels and calculated as 2^-[(Ct of gene) – (Ct of GAPDH)]^, where Ct represents the threshold cycle for each transcript. The real-time PCR utilized the following primers: the ANXA3 forward and reverse primers were 5-CCCATCAGTGGA TGCTGAAG-3 and 5-TCACTAGGGCCACC ATGAGA-3, E-cadherin forward and reverse primers were TGCCCAGAAAATGAAAAAGG and GTGTATGTGGCAATGCGTTC;Vimentin forward and reverse primers were GAGAACTTTGCCGTTGAAGC and GCTTCCTGTAG GTGGCAATC**;** GAPDH forward and reverse primers were 5-TG ACCCAGATATGTTTGAG-3 and 5-CGTACAGGGATAGCACAG-3, respectively.

### Western blotting

The GC samples, including tumor and adjacent to tumor tissues, as well as cell lines, were lysed in RIPA lysis buffer, and the lysates were harvested by centrifugation (12,000rpm) at 4°C for 20 min. Approximately 30μg protein samples were then resolved in a 12% sodium dodecyl sulfate polyacrylamide gel for electrophoresis and transferred to a PVDF membrane. After blocking the non-specific binding sites for 60 min with 8% non-fat milk, the membranes were incubated overnight at 4°C with anti-human antibodies to ANXA3(1:1,000; OriGene), anti-human antibodies to E-cadherin, β-catenin, vimentin, fibronetin, snail, slug (1:1,000; Cell Signaling Technology), or anti-GAPDH (at 1:10,000; Abcam). The membranes were then washed four times with TBST (tris-buffered saline with tween-20) for 10 min. After washing, the membranes were probed with the horseradish peroxidase-conjugated secondary antibody and visualized with chemiluminescent system (Cell signal, Danvers, USA). The band intensity was measured by the Quantity One software (Bio-Rad Laboratories, Inc. Hercules, CA, USA).

### Proliferation assay

Cell growth rate was detected by MTS cell proliferation assay. Cells were seeded in 96-well plates at a density of 5×10^2^ per well. The cell growth rate was detected using cell proliferation MTS kit according to the manufacturer's instruction (Promega, USA). Three independent experiments were performed.

### Colony formation assay

Cells were seeded in a 6-well plate at a density of 5×10^2^ cells per well. After 12 days of cultivation, surviving colonies (>50 cells per colony) were counted with 0.5% (m/v) crystal violet staining. Colony-forming efficiency (CFE %) was defined as the ratio of the number of colonies formed in culture to the number of cells inoculated. Three independent experiments were performed. Statistical analyses were carried out using the two-tailed unpaired Student's t-test.

### Cell invasion and migration assay

The Matrigel invasion assay was performed in a Transwell consisting polycarbonate membrane inserts with 8-μm pores (Corning, Shanghai, China) placed in a 24-well plate. The bottom of Transwell was coated with a thin layer of 0.5 mg/ml Matrigel Basement Membrane Matrix (BD Biosciences, Bedford, MA). Cells in 100 μl of RPMI 1640 without FBS were added in the Transwell, and 0.5 ml of RPMI 1640 containing 20% FBS was placed in the lower chamber. The cells were incubated at 37°C and allowed to invade through the Matrigel layer. After 48 hours, was fixed with 75% methanol for 10 minutes. The invaded cells on the lower surface of Transwell membranes were stained with 0.5% crystal violet for 1 hour. The stained cells were counted at 10 random fields under an inverted microscope. The migration assay was performed similarly as invasion assay, except that Transwell membrane was coated with a thin layer of Matrigel Basement Membrane Matrix. Each experiment was performed in triplicate. Statistical analyses were carried out using the two-tailed unpaired Student’ s t-test.

### Statistical analysis

Statistical analyses were performed with SPSS 17.0 software (SPSS, Chicago, Illinois, USA). An independent-sample *t* test was used to detect significant differences between continuous variables. The chi-squared test was used to analyze the relationships between ANXA3expression and various clinicopathological parameters. Survival curves were calculated using the Kaplan–Meier method and compared by the log-rank test. Univariate and multivariate survival analyses were performed by Cox proportional hazards regression model. The results were expressed as mean ± SD. Differences were considered significant at P<0.05.

## Abbreviations

gastric cancer: GC
epithelial–mensenchymal transition: EMT
annexin A3: Anxa3
immunohistochemical: IHC

## References

[1] Jemal A, Bray F, Center MM, Ferlay J, Ward E, Forman D (2011). Global cancer statistics. CA: a cancer journal for clinicians.

[2] Hartgrink HH, Jansen EP, van Grieken NC, van de Velde CJ (2009). Gastric cancer. Lancet.

[3] Zheng Y, Wang DD, Wang W, Pan K, Huang CY, Li YF, Wang QJ, Yuan SQ, Jiang SS, Qiu HB, Chen YM, Zhang XF, Zhao BW (2014). Reduced expression of uroplakin 1A is associated with the poor prognosis of gastric adenocarcinoma patients. PloS one.

[4] Wang DD, Chen YB, Pan K, Wang W, Chen SP, Chen JG, Zhao JJ, Lv L, Pan QZ, Li YQ, Wang QJ, Huang LX, Ke ML (2012). Decreased expression of the ARID1A gene is associated with poor prognosis in primary gastric cancer. PloS one.

[5] Sekhon K, Bucay N, Majid S, Dahiya R, Saini S (2016). MicroRNAs and epithelial-mesenchymal transition in prostate cancer. Oncotarget.

[6] Miao L, Huang Z, Zengli Z, Li H, Chen Q, Yao C, Cai H, Xiao Y, Xia H, Wang Y (2016). Loss of long noncoding RNA FOXF1-AS1 regulates epithelial-mesenchymal transition, stemness and metastasis of non-small cell lung cancer cells. Oncotarget.

[7] Mlcochova H, Machackova T, Rabien A, Radova L, Fabian P, Iliev R, Slaba K, Poprach A, Kilic E, Stanik M, Redova-Lojova M, Svoboda M, Dolezel J, Vyzula R, Jung K, Slaby O (2016). Epithelial-mesenchymal transition-associated microRNA/mRNA signature is linked to metastasis and prognosis in clear-cell renal cell carcinoma. Scientific reports.

[8] Guo J, Fu Z, Wei J, Lu W, Feng J, Zhang S (2015). PRRX1 promotes epithelial-mesenchymal transition through the Wnt/beta-catenin pathway in gastric cancer. Medical oncology.

[9] Huang J, Xiao D, Li G, Ma J, Chen P, Yuan W, Hou F, Ge J, Zhong M, Tang Y, Xia X, Chen Z (2014). EphA2 promotes epithelial-mesenchymal transition through the Wnt/beta-catenin pathway in gastric cancer cells. Oncogene.

[10] Cardenas H, Vieth E, Lee J, Segar M, Liu Y, Nephew KP, Matei D (2014). TGF-beta induces global changes in DNA methylation during the epithelial-to-mesenchymal transition in ovarian cancer cells. Epigenetics.

[11] Zhang J, Shao X, Sun H, Liu K, Ding Z, Chen J, Fang L, Su W, Hong Y, Li H, Li H (2016). NUMB negatively regulates the epithelial-mesenchymal transition of triple-negative breast cancer by antagonizing Notch signaling. Oncotarget.

[12] Zhang J, Wen X, Ren XY, Li YQ, Tang XR, Wang YQ, He QM, Yang XJ, Sun Y, Liu N, Ma J (2016). YPEL3 suppresses epithelial-mesenchymal transition and metastasis of nasopharyngeal carcinoma cells through the Wnt/beta-catenin signaling pathway. Journal of experimental & clinical cancer research.

[13] Perron B, Lewit-Bentley A, Geny B, Russo-Marie F (1997). Can enzymatic activity, or otherwise, be inferred from structural studies of annexin III?. The Journal of biological chemistry.

[14] Gerke V, Moss SE (2002). Annexins: from structure to function. Physiological reviews.

[15] Rescher U, Gerke V (2004). Annexins--unique membrane binding proteins with diverse functions. Journal of cell science.

[16] Zhai JM, Sun SJ, Wang W, Zeng C (2014). Expression of annexin A3 in gastric cancer and its correlation with proliferation and apoptosis. Asian Pacific journal of cancer prevention.

[17] Park JE, Lee DH, Lee JA, Park SG, Kim NS, Park BC, Cho S (2005). Annexin A3 is a potential angiogenic mediator. Biochemical and biophysical research communications.

[18] Jung EJ, Moon HG, Park ST, Cho BI, Lee SM, Jeong CY, Ju YT, Jeong SH, Lee YJ, Choi SK, Ha WS, Lee JS, Kang KR, Hong SC (2010). Decreased annexin A3 expression correlates with tumor progression in papillary thyroid cancer. Proteomics Clinical applications.

[19] Yan X, Yin J, Yao H, Mao N, Yang Y, Pan L (2010). Increased expression of annexin A3 is a mechanism of platinum resistance in ovarian cancer. Cancer research.

[20] Liu YF, Xiao ZQ, Li MX, Li MY, Zhang PF, Li C, Li F, Chen YH, Yi H, Yao HX, Chen ZC (2009). Quantitative proteome analysis reveals annexin A3 as a novel biomarker in lung adenocarcinoma. The Journal of pathology.

[21] Niimi S, Harashima M, Gamou M, Hyuga M, Seki T, Ariga T, Kawanishi T, Hayakawa T (2005). Expression of annexin A3 in primary cultured parenchymal rat hepatocytes and inhibition of DNA synthesis by suppression of annexin A3 expression using RNA interference. Biological & pharmaceutical bulletin.

[22] Yip KT, Das PK, Suria D, Lim CR, Ng GH, Liew CC (2010). A case-controlled validation study of a blood-based seven-gene biomarker panel for colorectal cancer in Malaysia. Journal of experimental & clinical cancer research.

[23] Pan QZ, Pan K, Wang QJ, Weng DS, Zhao JJ, Zheng HX, Zhang XF, Jiang SS, Lv L, Tang Y, Li YQ, He J, Liu Q, Chen CL, Zhang HX, Xia JC (2015). Annexin A3 as a potential target for immunotherapy of liver cancer stem-like cells. Stem cells.

[24] Pan QZ, Pan K, Weng DS, Zhao JJ, Zhang XF, Wang DD, Lv L, Jiang SS, Zheng HX, Xia JC (2015). Annexin A3 promotes tumorigenesis and resistance to chemotherapy in hepatocellular carcinoma. Molecular carcinogenesis.

[25] Wu N, Liu S, Guo C, Hou Z, Sun MZ (2013). The role of annexin A3 playing in cancers. Clinical & translational oncology.

[26] Bianchi C, Bombelli S, Raimondo F, Torsello B, Angeloni V, Ferrero S, Di Stefano V, Chinello C, Cifola I, Invernizzi L, Brambilla P, Magni F, Pitto M, Zanetti G, Mocarelli P, Perego RA (2010). Primary cell cultures from human renal cortex and renal-cell carcinoma evidence a differential expression of two spliced isoforms of Annexin A3. The American journal of pathology.

[27] Wu N, Liu S, Guo C, Hou Z, Sun MZ (2013). The role of annexin A3 playing in cancers. Clinical & translational oncology.

[28] Baine MJ, Chakraborty S, Smith LM, Mallya K, Sasson AR, Brand RE, Batra SK (2011). Transcriptional profiling of peripheral blood mononuclear cells in pancreatic cancer patients identifies novel genes with potential diagnostic utility. PloS one.

[29] Kollermann J, Schlomm T, Bang H, Schwall GP, von Eichel-Streiber C, Simon R, Schostak M, Huland H, Berg W, Sauter G, Klocker H, Schrattenholz A (2008). Expression and prognostic relevance of annexin A3 in prostate cancer. European urology.

[30] Tong M, Fung TM, Luk ST, Ng KY, Lee TK, Lin CH, Yam JW, Chan KW, Ng F, Zheng BJ, Yuan YF, Xie D, Lo CM, Man K, Guan XY, Ma S (2015). ANXA3/JNK Signaling Promotes Self-Renewal and Tumor Growth, and Its Blockade Provides a Therapeutic Target for Hepatocellular Carcinoma. Stem cell reports.

